# Supplementary figures and images for: Characterizing Muscle Tissue Quality Post-Stroke: Echovariation as a Clinical Indicator
Source: J Clin Med. 2024 Dec 20;13(24):7800. doi: 10.3390/jcm13247800 (PMC11728361; doi:10.3390/jcm13247800)

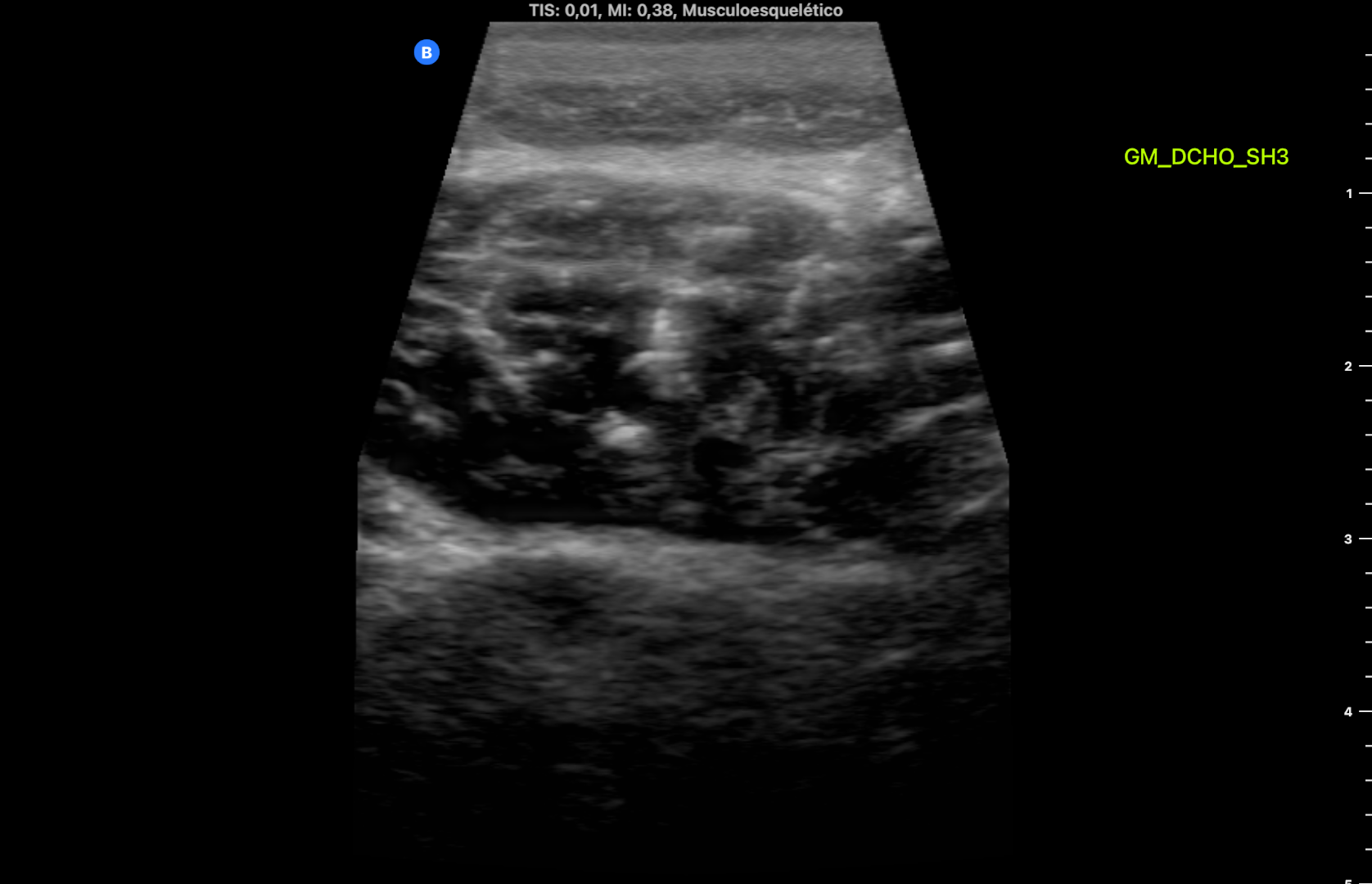

Supplement: Supplementary file 1 [file jcm-13-07800-s001.zip › Suppl. Figure 1.png]

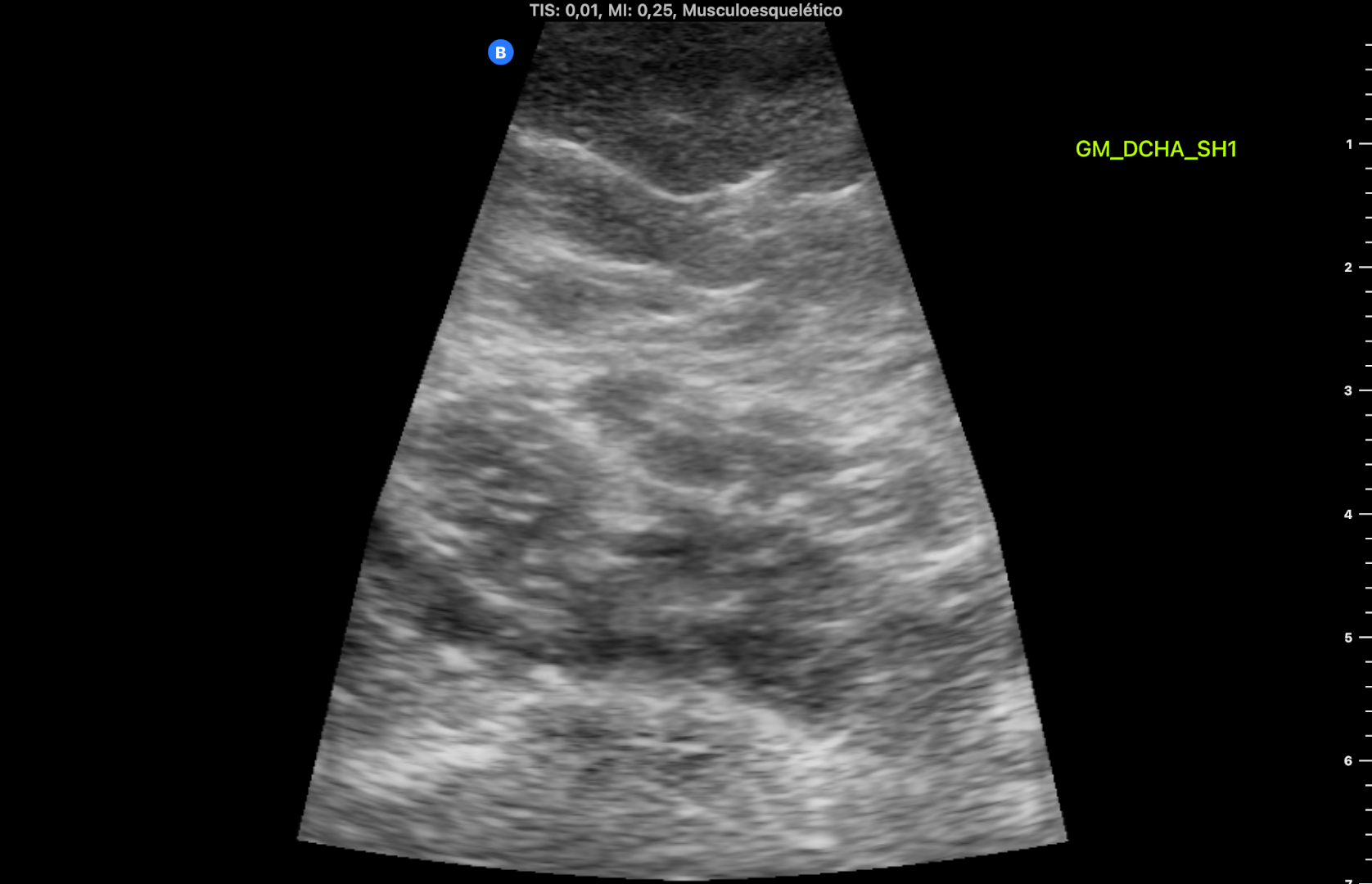

Supplement: Supplementary file 1 [file jcm-13-07800-s001.zip › Suppl. Figure 2.png]

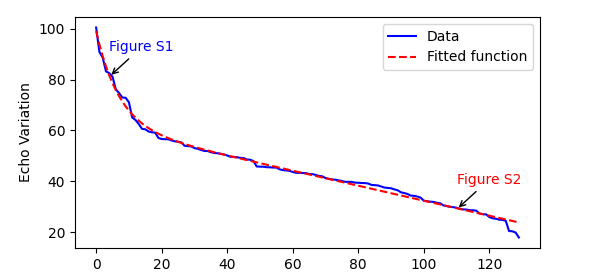

Supplement: Supplementary file 1 [file jcm-13-07800-s001.zip › Suppl. Figure 3.png]

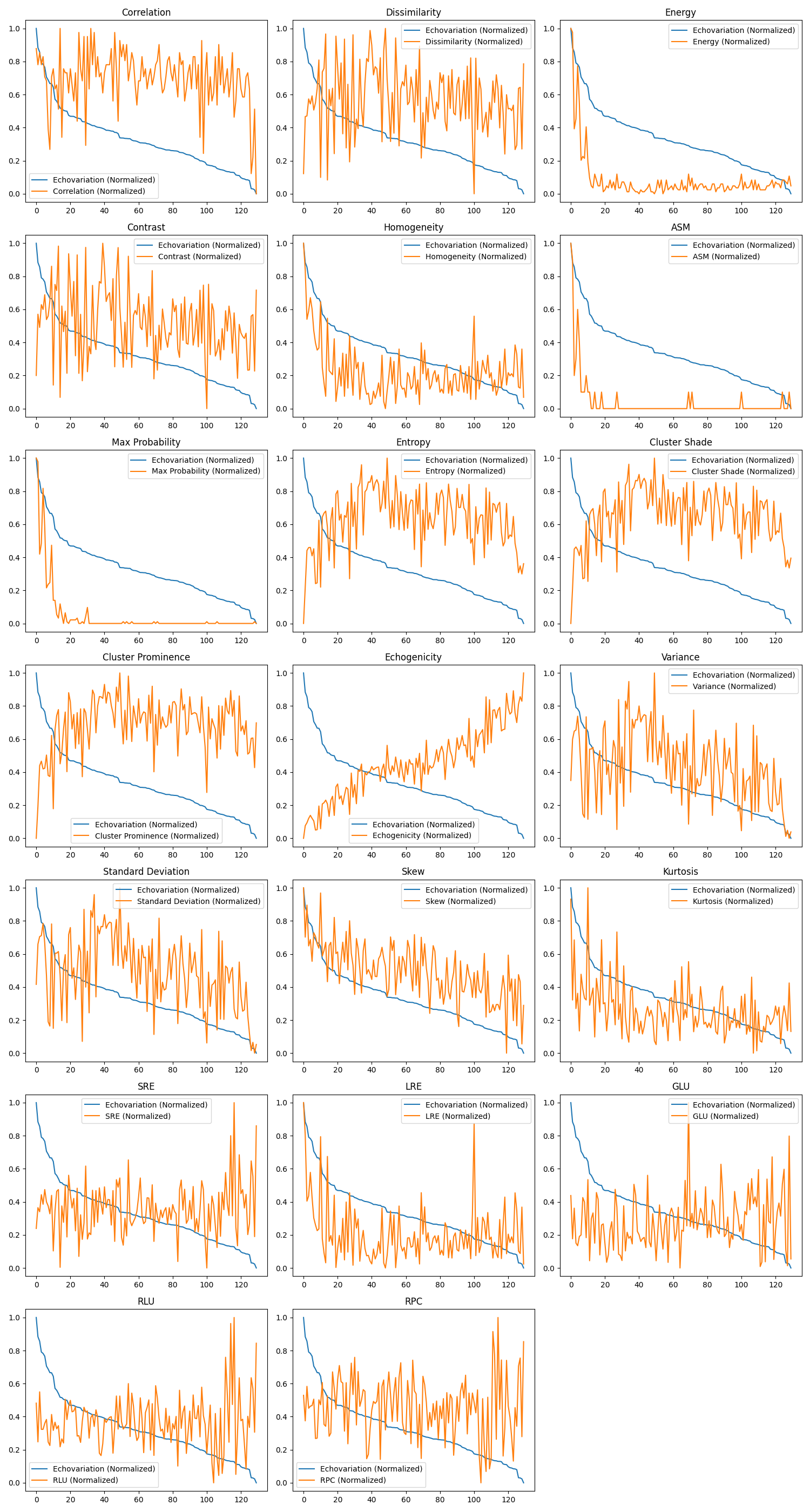

Supplement: Supplementary file 1 [file jcm-13-07800-s001.zip › Suppl. Figure 4.png]

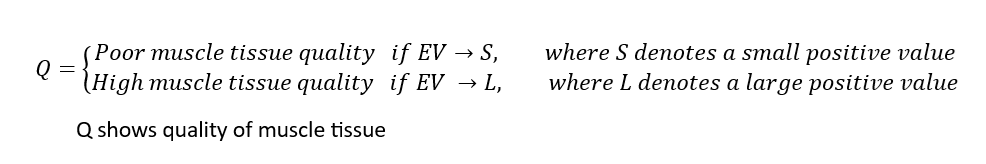

Supplement: Supplementary file 1 [file jcm-13-07800-s001.zip › Suppl. Figure 5.png]
